# Leadless pacemaker implantation in a patient with allergy to chromium, cobalt, titanium, and nickel

**DOI:** 10.1016/j.hrcr.2024.11.011

**Published:** 2024-11-22

**Authors:** José M. Sánchez-Moreno, Torcuato Garrido-Arroquia Jurado, Manuel Molina-Lerma, Rosa Macías Ruiz, Pablo J. Sánchez Millán, Miguel Álvarez

**Affiliations:** Arrythmia Unit, Cardiology Department, Hospital Universitario Virgen de las Nieves, Granada, Spain

**Keywords:** Leadless, Pacemaker, AV block, Metal allergies, Complications

## Introduction

Allergic reaction to the components of cardiac implantable electronic devices such as permanent pacemakers is rare. A classic solution to this problem is the implantation of a device covered in hypoallergenic material or that does not contain the identified allergen. Leadless pacemakers have been developed to address limitations typically related to pulse generator pocket and transvenous leads of conventional pacemaker systems. We report the first case of a patient with known multiple metal allergies who underwent leadless pacemaker implantation.

**Key Teaching Points**

• Allergic reactions to multiple pacemaker compounds are extremely rare but cause mild to severe symptoms and may even require removal of the device.
• Infection of the pacemaker pocket must be ruled out as part of the differential diagnosis.
• Pacing with entirely leadless devices is an emerging area of interest that addresses limitations typically related to pulse generator pocket and transvenous leads.
• Leadless pacing could be a safe alternative to conventional pacemakers in case of metal allergies.

## Case report

**Figure 1:**
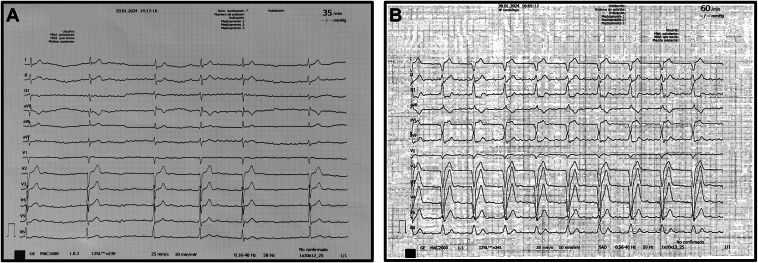
Basal 12-lead electrocardiogram with atrial fibrillation with slow ventricular response (**A**) and paced after leadless pacemaker implantation (**B**).

**Figure 2:**
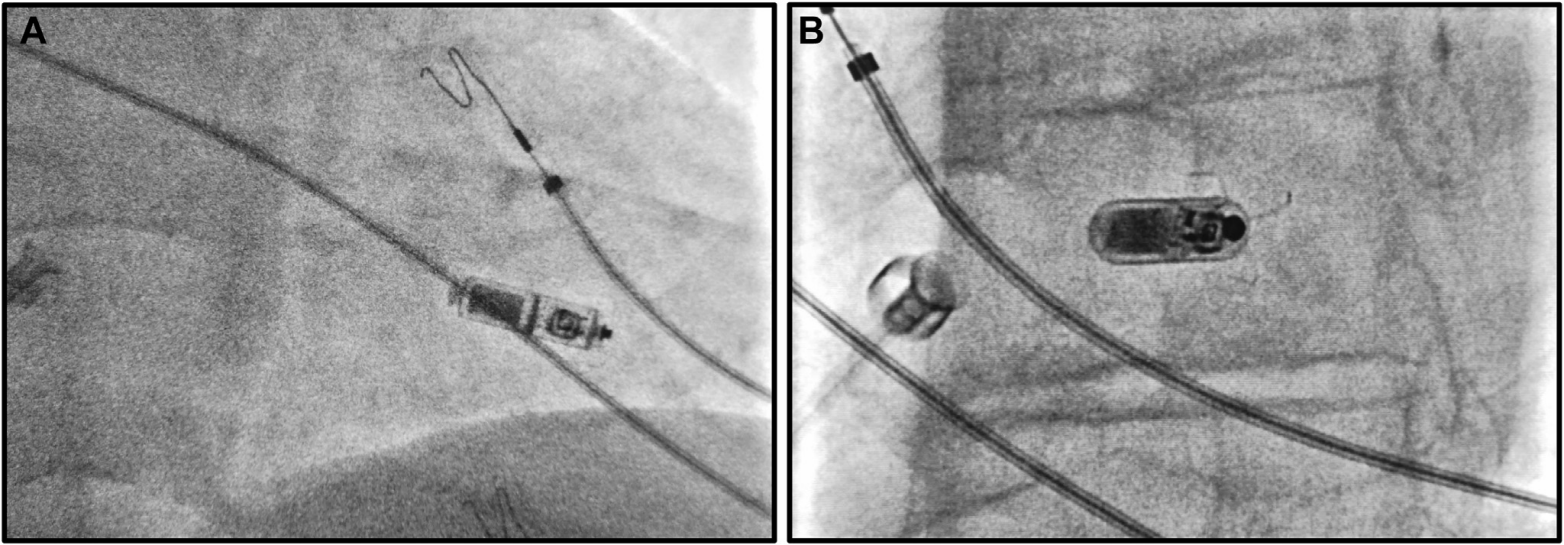
Right (**A**) and left (**B**) anterior oblique fluoroscopic views demonstrating the leadless device (Micra®) placed in the mid septum.

**Figure 3:**
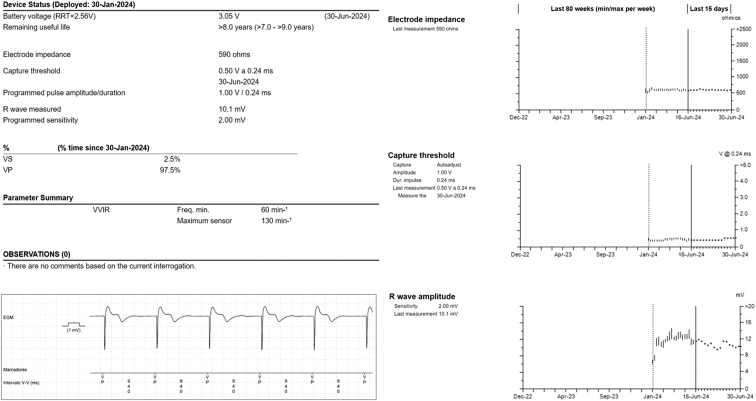
Remote monitoring at 6-month follow-up.

A 73-year-old man with a history of hypertension, dyslipidemia, former smoker, typical atrial flutter (cavotricuspid isthmus ablation in June 2016) and permanent atrial fibrillation with slow ventricular response was admitted to our hospital in January 2024 for symptomatic atrioventricular block (Figure 1A). In December 2017, he had been referred for dermatologic investigations including epicutaneous patch testing, oral metal challenge, and lymphocyte-stimulating test. It was concluded that he had allergies to potassium dichromate, cobalt chloride, titanium, and nickel sulfate. Given the difficulty in obtaining a gold-coated pacemaker in a short period of time and the fact that nickel, cobalt, and chromium are components present in pacemaker leads (Alloy MP35N®) that could be exposed in case of insulation deterioration, the implantation of a leadless pacemaker was considered. Because the allergic reaction may depend on the amount of material exposed to body tissues or fluids, the implantation of a leadless device -Micra™ Transcatheter Pacing System (Medtronic, Minneapolis, MN) was chosen. The first mid-septal implantation attempt was successfully performed (Figure 2) with satisfactory acute sensing (6.5 mV), impedance (595 Ohms), and threshold (0.50 V @ 0.24 ms) measurements (Figures 1B and 3). At 6-month follow-up, no complications or symptoms were reported related to the implantation procedure. Remote monitoring collected information from the device and indicated all pacemaker system values remained stable, within the recommended ranges (Figure 3).

## Discussion

Allergic reactions to pacemaker compounds are extremely rare, but multiple pacemaker component allergies are known to exist.^1^, ^2^, ^3^ Reported allergens are titanium,^1^ silicone,^1^ nickel,^4^ mercury,^5^ chromate,^5^ epoxy resin,^6^ polyurethane,^7^ cadmium,^8^ and cobalt, among others. Once infection has been excluded, allergy should be considered in the differential diagnosis. The appearance of erythema or eczema over the pacemaker area accompanied by local inflammation can be the first sign, but in severe cases, exteriorization of the device can occur. Some treatments for pacemaker-induced allergic reactions are described in various case reports.^9^^,^^10^ A common alternative is the implantation of a device that does not contain the identified allergen or 1 that is covered in hypoallergenic material (gold-plated, polytetrafluoroethylene bag, silicone coating).^11^, ^12^, ^13^

Pacing with entirely leadless devices is an emerging area of interest. The primary advantage of a leadless pacemaker is the elimination of several complications associated with transvenous pacemakers and leads—pocket infections, hematoma, lead dislodgement, and lead fracture. The leadless pacemaker also has cosmetic appeal because there is no chest incision or visible pacemaker pocket.^14^ According to current guidelines, leadless pacemakers should be considered as an alternative to transvenous pacemakers when no upper extremity venous access exists or when risk of device pocket infection is particularly high, such as previous infection and patients on hemodialysis.^15^ However, its use in cases of allergies to conventional pacemaker components has not been described.

In the case of Micra™, the system is made of titanium, but it is coated with a layer of parylene. Therefore, the only allergenic surfaces in contact with body tissues or fluids would be the electrode area and the self-expanding nitinol (an alloy of titanium and nickel) tines, and the amount of titanium or nickel that could leak from the device would be extremely low.

Cognizant of the fact that the complete removal of the allergenic material is usually mandatory when an allergic reaction occurs and that it would be especially challenging in the case of pacemaker dependency, we consider that leadless pacemakers could be an alternative option, because of their feasibility and greater availability.

## Disclosures

None declared.
